# Advances in Sensors-Centric Microprocessors and System-on-Chip

**DOI:** 10.3390/s120404820

**Published:** 2012-04-12

**Authors:** Juan A. Gómez-Pulido, Miguel A. Vega-Rodríguez

**Affiliations:** Department of Technologies of Computers and Communications, Polytechnic School, University of Extremadura, Campus Universitario s/n, 10003 Caceres, Spain; E-Mail: mavega@unex.es

Sensors-based systems are nowadays an extended technology for many markets due to their great potential in the collection of data from the environment and the processing of such data for different purposes. A typical example is the wireless sensor devices, where the outer temperature, humidity, luminosity and many other parameters can be acquired, measured and processed in order to build useful and fascinating applications that contribute to human welfare. In this scenario, the processing architectures of the sensors-based systems play a very important role. The requirements that are necessary for many such applications (real-time processing, low-power consumption, reduced size, reliability, security and many others) means that research on advanced architectures of Microprocessors and System-on-Chips (SoC) is needed to design and implement a successful product. In this sense, there are many challenges and open questions in this area that need to be addressed.

Along this line, the special issue “Microprocessors and System-on-Chip” of *Sensors* journal seeks to explore the latest advances in sensors-centric theoretical or practical aspects, such as: microprocessors, SoC, real-time processing, reconfigurable architectures and field programmable gate arrays (FPGAs), low-power devices, operating systems, interfaces and protocols, compilers and software tools, performance analysis, and applications and case studies, among others. For this purpose, of 25 manuscripts received, 11 original and high quality manuscripts were selected to be included in the special issue. The authors of the selected manuscripts belong to universities in China, Italy, Korea, Mexico, Portugal, Spain and Taiwan. Each manuscript was reviewed by between two and four reviewers—prestigious researchers in the same topics as the articles—and underwent up to three rounds of peer-review.

The first article [1] in this special issue is entitled “Network Coding on Heterogeneous Multi-Core Processors for Wireless Sensor Networks”, by Deokho Kim, Karam Park and Won W. Ro. This article explains the development of an efficient algorithm to reduce the computational cost and complexity of the network coding in wireless sensor networks using heterogeneous multi-core processors, in view of the future use of high performance architectures as processing nodes of the wireless sensor networks.

The second article [2] is entitled “Real-Time Telemetry System for Amperometric and Potentiometric Electrochemical Sensors”, by Wei-Song Wang, Hong-Yi Huang, Shu-Chun Chen, Kuo-Chuan Ho, Chia-Yu Lin, Tse-Chuan Chou, Chih-Hsien Hu, Wen-Fong Wang, Cheng-Feng Wu and Ching-Hsing Luo. In this article the authors propose a real-time telemetry system for amperometric and potentiometric electrochemical sensors. The authors use the reconfigurable hardware technology based on FPGA to implement a microcontroller unit, because this technology provides a significant reduction to the power consumption. The developed system includes a graphical user interface to display data in real-time, an analog-to-digital converter, a radio frequency transceiver, and readout circuits, which include a potentiostat and an instrumentation amplifier. As a result, the system has high linearity, small size, high portability, and high integration.

The third article [3] is “Historical Building Monitoring Using an Energy-Efficient Scalable Wireless Sensor Network Architecture”, by Juan V. Capella, Angel Perles, Alberto Bonastre and Juan J. Serrano. The research exposed in this article stems from the need for a method for early detection of pests in wooden masterpieces and historical buildings. For this purpose, a set of wireless sensor nodes has been designed in order to facilitate the scalability and reliability by means of a cluster-based dynamic-tree hierarchical Wireless Sensor Network, following two important goals: minimizing the economic cost of the whole system and maximizing power saving of the nodes. The strategy followed by the authors involves adapting the hardware of some nodes to provide better antennas.

The fourth article [4] is “An LDPC Decoder Architecture for Wireless Sensor Network Applications”, by Andrea Dario Giancarlo Biroli, Maurizio Martina and Guido Masera. This article elaborates on the use of the hardware of wireless sensors for an important timely topic: energy efficiency. For this purpose, the authors consider coded communication between a couple of wireless sensor devices as a method to reduce the dissipated energy per transmitted bit with respect to uncoded communication, obtaining high power savings in the range of 40–80%. For coded communication, some Different Low Density Parity Check (LDPC) codes are considered.

The fifth article [5] is entitled “Fast Decision Algorithms in Low-Power Embedded Processors for Quality-of-Service Based Connectivity of Mobile Sensors in Heterogeneous Wireless Sensor Networks”, by María D. Jaraíz-Simón, Juan A. Gómez-Pulido, Miguel A. Vega-Rodríguez and Juan M. Sánchez-Pérez. The authors tackle the problem where a mobile wireless sensor discovers multiple heterogeneous wireless sensor networks at the same time, having to decide the best network to connect to, according to the quality of service (QoS) characteristics. This optimization problem considers a fitness function to adjust a set of QoS weights. The high computational effort required leads to the development of a fast decision algorithm to be performed in a SoC architecture where a custom embedded reconfigurable processor based on FPGA is designed to achieve several goals necessary for competitive mobile terminals: good performance, low power consumption, low economic cost, and small area integration.

The sixth article [6] is entitled “A Web Service-Based Framework Model for People-Centric Sensing Applications Applied to Social Networking”, by David Nunes, Thanh-Dien Tran, Duarte Raposo, André Pinto, André Gomes and Jorge Sá Silva. This interesting article uses hardware and architectures to establish a relationship between sensors and social networks like Facebook, sharing mobile phones and sensors, enabling the creation of people-centric sensing systems. The authors have developed a Web Service to provide solutions such as the detection of users' activities and locations, sharing this information amongst the user's friends within a social networking site. This research deserves to be recognized due to the level of interest in this topic. The prototype of the architecture developed represents a first step into a research on people-centric sensing, where future advances of SoC will be important for the development of sensors specifically oriented for these kinds of systems.

The seventh article [7] is “Parametric Dense Stereovision Implementation on a System-on Chip (SoC)”, by Alfredo Gardel, Pablo Montejo, Jorge García, Ignacio Bravo and José L. Lázaro. This article proposes a SoC implementation of a dense recovery of stereovision 3D measurements. As vision sensors have an increasing number of pixels, the FPGA-based SoC designed for stereovision provides a scalable architecture, high performance and real-time processing of stereo image flow. Some characteristics of this architecture are: double buffering techniques, pipelined processing, and no external memory. The authors point out that this development can be applied in autonomous systems, acting as a coprocessor to reconstruct 3D images with high density information in real time.

The eighth article [8] is entitled “Dual Super-Systolic Core for Real-Time Reconstructive Algorithms of High-Resolution Radar/SAR Imaging Systems”, by Alejandro Castillo Atoche and Javier Vázquez Castillo. The authors have addressed the design of a high-speed dual super-systolic array core for the enhancement/reconstruction of remote sensing imaging of radar/synthetic aperture radar sensor systems. The selected reconstructive signal processing algorithms are parallelized and mapped into a high performance embedded computing architecture based on FPGA platforms. The proposed architecture has tested solving the nonlinear ill-posed inverse problem of nonparametric estimation of the power spatial spectrum pattern from a remotely sensed scene, achieving the real-time requirements.

The ninth article [9] is “Using SRAM Based FPGAs for Power-Aware High Performance Wireless Sensor Networks”, by Juan Valverde, Andres Otero, Miguel Lopez, Jorge Portilla, Eduardo de la Torre and Teresa Riesgo. The authors propose the use of FPGA-based microprocessors to satisfy the increasing computing requirements of wireless sensor nodes, instead of traditional ultra-low power microcontrollers with limited computing power. The commitment to use FPGA devices is based on the benefit of reduced execution time. The authors demonstrate this by proposing an innovative FPGA-based wireless sensor node architecture, taking advantage of the parallelism and partial reconfiguration capabilities, and configuring a power-aware management system for energy saving.

The tenth article [10] is entitled “Distributed Coding/Decoding Complexity in Video Sensor Networks”, by Paulo J. Cordeiro and Pedro Assunção. This article addresses the most relevant challenges posed by Video Sensor Networks, a recent communication infrastructure used to capture and transmit dense visual information from an application context. The authors propose a novel Video Sensor Network architecture where large sets of visual sensors with embedded processors are used for compression and transmission of coded streams to gateways. A method to reduce the decoding complexity is proposed to operate at the transcoding gateway whenever decoders with constrained resources are targeted.

The eleventh article [11] is entitled “An Advanced Compiler Designed for a VLIW DSP for Sensors-Based Systems”, by Xu Yang and Hu He. The authors propose an advanced custom compiler designed for a Very Long Instruction Word (VLIW) Digital Signal Processor (DSP) architecture, ready to provide computation power and energy efficiency advantages, which satisfies the requirements of sensor-based systems. The performance results obtained match that of the current state-of-the-art DSP processors.
